# A new treatment for eating disorders combining physical exercise and dietary therapy (the PED-t): experiences from patients who dropped out

**DOI:** 10.1080/17482631.2020.1731994

**Published:** 2020-02-21

**Authors:** Maria Bakland, Jan H. Rosenvinge, Rolf Wynn, Venke Sørlie, Jorunn Sundgot-Borgen, Therese Fostervold Mathisen, Tove Aminda Hanssen, Franziska Jensen, Kjersti Innjord, Gunn Pettersen

**Affiliations:** a Department of Health and Care Science, UiT - The Artic University of Norway, Tromsø, Norway; b Department of Psychology, UiT - The Artic University of Norway, Tromsø, Norway; c Department of Clinical Medicine, UiT - The Artic University of Norway, Tromsø, Norway; d Division of Addictions and Mental Health, University Hospital of North Norway, Tromsø, Norway; e Center for clinical nursing research, Lovisenberg Deaconal University College, Oslo, Norway; f Department of Sports Medicine, Norwegian School of Sport Sciences, Oslo, Norway; g Faculty of Health and Welfare,Østfold University College, Fredrikstad, Norway; h Department of Heart Disease, University Hospital of North Norway, Tromsø, Norway; i Department of Language and Culture, UiT - The Artic University of Norway, Tromsø, Norway; j Department of Education, UiT - The Artic University of Norway, Tromsø, Norway; k The Eating Disorder Association “Spisfo”, Tromsø, Norway

**Keywords:** Eating disorders, treatment, qualitative analysis, patient experiences, dropout

## Abstract

**Purpose**: Eating disorders (ED) are complex and severe illnesses where evidence-based treatment is needed to recover. However, about half of the patients with ED do not respond to treatments currently available, which call for efforts to expand the portfolio of treatments. The aim of this study was to explore experiences from patients who dropped out of a new treatment for bulimia nervosa and binge ED, combining **p**hysical **e**xercise and **d**ietary **t**herapy (PED-t).

**Methods**: We conducted open-ended face-to-face interviews. The interviews were transcribed verbatim and the data were analysed with a phenomenological hermeneutical approach.

**Results**: Three themes emerged: “standing on the outside”, “unmet expectations” and “participation not a waste of time”. Feelings of standing on the outside were elicited by being different from other group members and having challenges with sharing thoughts. Unmet expectations were related to treatment content and intensity, as well as the development of unhealthy thoughts and behaviours. Finally, some positive experiences were voiced.

**Conclusion**: A need to clarify pre-treatment expectations and refining criteria for treatment suitability is indicated. The findings have contributed to the chain of clinical evidence regarding the PED-t and may lead to treatment modifications improving the treatment and thereby reducing drop out.

## Introduction

Family-based therapy and cognitive-behavioural therapy (CBT) are recommended in several treatment guidelines worldwide and appear as generally accepted evidence-based treatments for eating disorders (ED). However, about half of the patients with ED fail to respond to such treatments (Linardon & Wade, 2018; Wilson, Grilo, & Vitousek, 2007).

Considering the severity of ED, these findings call for efforts to include new treatments in the portfolio of evidence-based treatments. A second reason to increase the portfolio of treatments refers to the diversity of factors that maintain ED or that may help in the recovery process. As far as general societal health is concerned, new treatments should address the most prevalent EDs, i.e., bulimia nervosa (BN) and binge eating disorder (BED) (Rosenvinge & Pettersen, 2015).

A new treatment for patients with BN and BED combining physical exercise and dietary therapy (PED-t) has been developed (Mathisen et al., 2017). The PED-t rests on a conceptual model positing the beneficial effects of physical activity for improving mental health (Lubans et al., 2016). Such effects are documented in the treatment of several mental illnesses, notably depression (Josefsson, Lindwall, & Archer, 2014; Rosenbaum, Tiedemann, & Ward, 2014) and anxiety (Jayakody, Gunadasa, & Hosker, 2013). As for EDs, dietary consultations have promoted an early change in eating routines and enhanced outcomes from established treatment modules (Hsu et al., 2001; Painot, Jotterand, Kammer, Fossati, & Golay, 2001), also for BN (Sundgot-Borgen, Rosenvinge, Bahr, & Schneider, 2002). A randomized controlled trial (RCT) conducted in Norway that framed the present study has so far shown that the PED-t performs equally effective as CBT in alleviating BN and BED symptoms both immediately and at a one-year follow-up (Mathisen, 2018).

There may be a disparity between a statistically significant effect and the patients’ experiences of a given treatment. This is an important reason why exploring patients’ treatment experiences is generally recognized as part of clinical evidence (Richards & Hallberg, 2015). Accordingly, previous studies have explored treatment experiences among the patients who completed the PED-t (Bakland et al., 2019; Pettersen et al., 2017) as well as their pre-treatment expectations (Pettersen et al., 2019). No disparity was detected between the statistically significant effects and their general positive experiences. Overall, the PED-t stands out as helpful in promoting recovery from BN and BED. Importantly, patients’ experiences echoed the experiences reported by the PED-t therapists (Bakland et al., 2018). However, some patients wished a more flexible time-frame to allow booster sessions, and voiced feelings of being “different” in terms of age, interests or the nature of their ED (Bakland et al., 2019; Pettersen et al., 2017). These findings were congruent with previous studies which explored treatment experiences with several treatment approaches to ED (Krautter & Lock, 2004; Lose et al., 2014; Money, Genders, Treasure, Schmidt, & Tchanturia, 2011; Poulsen, Lunn, & Sandros, 2010; Sánchez-Ortiz et al., 2011) in terms of overall positive experiences, yet with suggestions for improvements.

In RCT studies, intention-to-treat analyses, as well as dropout analyses, are important sources of knowledge about treatment efficacy. Qualitative studies should follow the same kind of logic and explore experiences among patients who drop out of treatment. Such studies are needed to capture more of the variety of experiences, and which may be under-reported in the traditional treatment-satisfaction studies (Pettersen et al., 2018). However, to our knowledge, no previous qualitative studies have specifically addressed ED patients who have dropped out of a treatment with the intention to explore their treatment experiences. To accommodate this need, the aim of the present study was to explore the experiences of patients who dropped out of the PED-t treatment.

## Methods

### Context and treatment

The study context was an RCT conducted between 2014–2016 that compared the PED-t treatment with CBT (Mathisen et al., 2017). During 4 months, both treatments were run in a 20-session group therapy format with 5–8 participants per group. The training programme in the PED-t treatment consisted of three weekly exercise sessions, each lasting 40–60 min. Two sessions were resistance exercise, of which physical trainers supervised one. The third session consisted of unsupervised interval running. Following a traditional pyramid structure, the running program comprised progressive interval periods and active rest periods. The dietary therapy was led by a dietician and included three psychoeducative modules “dietary routines and structure” (five sessions), “nutritional knowledge and practical skills” (12 sessions), and “summary and future plans” (three sessions). Further details about the nature and rationale of the treatment are provided elsewhere (Mathisen et al., 2017; Pettersen et al., 2018).

### User advisory group

A user advisory group may improve the relevance and quality of research (Oliver, Liabo, Stewart, & Rees, 2015). Accordingly, in the present study, two members from a national ED patient organization were included. In regular meetings with the research group, they shared their personal experiences with ED treatment and contributed to the understanding and analyses of the data as well as in the writing of the present paper.

### Participants

The principal investigator (JSB) contacted all 15 women who did not complete at least 80% of the treatment, with information about, and a request to participate in the present study. Informed consent from five participants was returned to the first author (MB). Their age ranged from 21 to 41 years. Three participants had BN, two had BED, and four reported a history of anorexia nervosa. One woman had children and lived with a partner. Three of them were studying at the university and two were employed. The women had completed between 2 and 10 of the 20 treatment sessions in total.

### Data collection

Face-to-face interviews were carried out at locations of the participants’ choice. The interviewer had a professional background as a nurse in the mental health field and no prior relations to the participants. Striving for openness towards the women’ experiences, they were encouraged to talk freely responding to one open-ended question: Can you (as one of those who left the PED-t) please tell me about your experiences with participating in the treatment? Follow-up questions were used to gain a deeper understanding of areas that seemed essential for each woman. The duration of the interviews ranged between 1 and 1.5 h and they were not repeated. To compensate for travel expenses and other costs, all participants received a gift card of NOK 250 (approximately USD 32). The audio-recorded interviews were transcribed verbatim.

### Data analysis

The consolidated criteria for reporting qualitative research (COREQ) (Tong, Sainsbury, & Craig, 2007) were used to promote explicit and comprehensive reporting of the research data. We performed an inductive, three-step data analysis inspired by a phenomenological hermeneutical approach and an interpretation theory of discourse (Lindseth & Norberg, 2004; Ricœur, 1976). This approach is suitable to explore lived experiences and involves a dialectic movement between the parts of the text and the text as a whole (Lindseth & Norberg, 2004). In the first step, a repetitive, *naïve reading of the whole* text was done to grasp an immediate understanding of the content and what it communicated. Next, a *structural analysis* divided the text into meaning units based on sentences or sections reflecting the research question and eventually themes disclosing meaning were formulated. The third step involved a *comprehensive understanding*. Here, the themes were summarized and reflected on in relation to the aim and context of the study, the authors’ pre-understandings, the naïve reading of transcripts, the structural analysis, and relevant literature (Lindseth & Norberg, 2004). All authors continuously monitored and discussed the analysis until consensus was reached about how to best interpret the data.

### Ethical considerations and data security

The RCT study was registered in the Clinical Trials registry (identifier: NCT02079935). The Norwegian Regional Committee for Medical and Health Research Ethics approved the present study (identifier: 2013/1871) based on the Helsinki declaration of informed consent, data security and the option to withdraw unconditionally upon request.

## Results

Three themes emerged from the analysis, reflecting experiences from those who dropped out from the PED-t. The themes were “standing on the outside”, “unmet expectations” and “participation not a waste of time”. Each theme is presented below along with subthemes and illustrative quotations from the interviews.

### Standing on the outside

#### Feeling different

A feeling of being different from other group members was evident, and the participants related their feelings of being different to life in general, age, interests, or being in other phases in terms of illness severity and recovery. One woman who had been ill for many years was afraid that the others’ inexperience would affect her benefits of participating:

I was the oldest and I have participated in treatment so many times in the past. The other women had never talked to anybody about their problem. I was afraid that I would fall back into the position of being the one who supports all the others (Participant 2).

A feeling of being different also emerged from listening to other participants talk about their overeating. One woman said:

Maybe I was not in the right target group. The amounts of food the other women talked about was unnatural for me. What I believe is overeating, might not be overeating after all (Participant 5).

Feeling different was furthermore related to previous experiences with physical exercise. Some participants were familiar with exercise and exercised daily before starting the treatment, whilst others had never exercised on a regular basis. These differences affected how the women experienced their benefit from participating in the treatment:

I had no problem talking to the other women, but they were just so different from me in terms of eating and exercising. I believe the treatment would have been more beneficial if the women in my group shared my interests, because then we might have talked about something relevant to me (Participant 4).

#### Having challenges with sharing thoughts

This subtheme was related to a lack of group atmosphere allowing participants to share mutual experiences in one united conversation. Rather, group discussions tended to evolve around one-to-one conversations between a group member and the therapist. In addition, some women occupied more time by talking than the other group members:

Three of the others in my group took a lot of space. Because of that, I often found myself in a listening position. I had a lot to say but I was never able to share my thoughts (Participant 1).

Perceived lack of experiences of exposing sensitive issues to others could worsen the communication with other group members:

When I came here for the first time, we sat down in a circle to talk about ourselves. This was a bit sudden to me, and I struggled and started crying. I dislike speaking to a group like that and I returned home with a headache (Participant 1).

### Unmet expectations

#### Needing more treatment intensity and support

The need for higher intensity and more support was generated from challenges in implementing the acquired knowledge in their daily life. One such challenge was to make good choices about meal compositions:

The dietary therapy was difficult because I had expected it to be more specific. Food has always been an issue for me and I felt that they did not go deep enough into the details and left me with too many choices. I wished there would have been a fixed plan for me to follow, on what to eat and when (Participant 3).

Other kinds of challenges were to harmonize the amount of foods and the level of physical exercise:

The therapists told me to exercise less and eat more. I wanted to go all in and do this, but I felt I was not receiving the support I needed. For me to be able to do that, I would have needed to talk to the therapists almost every day (Participant 4).

In addition, those who were unfamiliar with doing physical exercise found it challenging to comply with the exercise programme between each supervised treatment session:

There were too many days where I had to do everything by myself. Gradually I realized that I needed a more intensive treatment, like every other day. Then I might have been able to develop some good routines. When they asked me how I had done since we last met, I did not want to answer because I had not done so well (Participant 1).

Finally, unmet expectations were related to vague and imprecise requirements from the therapists:

The therapists’ guidance and suggestions were too vague for me. I need someone to be direct and strict, explaining me that I need to do this for myself, and that I need to work harder (Participant 3).

#### Lacking trust

Trusting the therapists’ advice seemed to be difficult for the participants. For instance, the dietary advice was experienced as too focused on rights and wrongs with little room for flexibility. Another challenge was believing in the sufficiency of the treatments exercise programme. A fear of gaining weight challenged the women’s ability to let go of their already established routines;

I was extremely preoccupied with losing weight and did not trust the advice they gave me. Of course, I was afraid to gain weigh by giving away control. Eating more and exercising less was not an alternative in my mind (Participant 4).

Lack of trust was also related to the programme and its rationale:

This experience has taught me that when you are ill enough to let your eating disorder run your daily life, you need something more than this treatment. Trying to follow advice on an ideal amount of food and exercise is not enough (Participant 5).

Lastly, lacking trust was experienced as the treatment was run outside a health service institution, and by therapists not obliged by a professional secrecy and code of ethics:

I have never told anyone that I have an ED earlier. You never know if they will keep their promise. I remember having a moment when it was my turn to talk and so I did. Afterwards I did not feel good about that. I guess I never felt completely safe in the treatment setting (Participant 5).

#### Developing unhealthy thoughts and behaviours

Unmet expectations were also generated by feelings that the programme elicited unhealthy thoughts and behaviours. The focus on nutrients and meals in the dietary therapy was experienced as stressful, particularly for those with a history of anorexia nervosa, and who had worked for a long time to eliminate such a focus. Despite the therapists’ advice, the preoccupation of calorie counting revived;

I downloaded the calorie-counting app that I had used on my phone earlier. I told myself that the app was not dangerous in itself. That is how the ball started rolling. Luckily, I was able to stop this unhealthy thinking early enough (Participant 1).

Being weighed at each treatment session was also challenging. Participants found themselves focusing on the weight results, which in return, triggered their weight and shape preoccupation;

We had to step on the weight each session, and I questioned this because I had been strongly advised the opposite when I was struggling with my anorexia. I decided it became too much of an unhealthy focus for me (Participant 1).

Finally, adding the treatments’ exercise programme on top of one’s own daily exercise routine led to a situation where one exercised too much. One woman reflected around her experience in the following way:

For each treatment session, I felt that something was wrong. I did not expect that the treatment would trigger my eating disorder, but I really became worse. I had expected an easier process and developing a healthier view on everything, but I ended up feeling a pressure to perform (Participant 5).

### Participation not a waste of time

Despite dropping out of the treatment, participants also voiced positive experiences and feelings of having utilized the potentials of the programme. In particular, some knowledge and tools were acquired. For example, it was beneficial to become more aware of their ED problem. Furthermore, participants experienced that the programme was helpful in regulating and normalizing the amount of physical exercise from being excessive or just to start exercising. Finally, there was a value in learning about the plate model and various nutrients needed in order to stay healthy:

I feel healthier now. I believe what I needed was some time and space after the treatment to let the knowledge mature. I still have days were I snap and eat too much, but now I have learned something about what too much really means (Participant 1).

## Discussion

This study reports on views from patients who dropped out from a new treatment for ED. Overall, patients who dropped out from the PED-t treatment experienced that they were different from other group members and felt that they stood outside. Important sources of such feelings were differences in symptom load, age, interests and life in general, as well as challenges with sharing thoughts. In addition, these patients reported some unmet expectations in terms of treatment content and intensity, and some described developing an unhealthy preoccupation with nutrients, physical exercise and weight. Finally, the patients experienced having gained some knowledge and tools and making use of this knowledge subsequently.

Feelings of standing on the outside are consistent with a previous study (Bakland et al., 2018) from our research group, and which explored experiences among the therapists who provided the PED-t. They reported that patients’ monopolization of group sessions jeopardized their efforts to create an including group climate. Challenges in establishing a mutual relationship between individuals in a treatment group are, however, generic, and not confined to ED or the PED-t (Hummelen, Wilberg, & Karterud, 2007) or to patients who actually completed the PED-t (Bakland et al., 2019). Such findings align with a recent meta-analysis (Burlingame, McClendon, & Yang, 2018) demonstrating that group cohesion contributes to outcomes across a variety of clinical conditions and therapeutic settings. Group-leaders in all theoretical orientations are therefore encouraged to foster cohesion. A further refinement of the PED-t should consider a stronger focus on selection criteria.

A plea for more treatment sessions and follow-up is a general finding which is also evident among other patient groups (Hummelen et al., 2007; Kerkelä, Jonsson, Lindwall, & Strand, 2015). However, the present results point to more specified issues, like the need for more support in implementing physical exercise and new dietary routines, as well as more support in the patients’ daily lives. Such issues align with other findings showing that experienced treatment benefits are linked to therapists’ availability and time to listen and understand the person behind the ED (Pettersen & Rosenvinge, 2002). In a further implementation of the PED-t program, a stronger focus on pre-treatment patient expectations may be important to address motivation.

The plea for more treatment sessions and therapist support could easily be complied for in future use of PED-t; however, the present study also revealed that the patients experienced the treatment as time-consuming and that the focus on nutrients and exercise caused stress. This aligns with previous findings suggesting that an important reason why patients drop out of treatment is to reduce the intensity of the treatment (Nordheim et al., 2018). Again, this may point to a need for discussing motivation for change and pre-treatment expectations. Moreover, having a history of anorexia nervosa seemed to have caused extra challenges with regards to nutrients and being weighted. This point reiterates the argument that the future implementation of the PED-t may need a stronger focus on treatment selection criteria. Such a variety of patient experiences represent a challenge when planning for treatments to fit individual needs.

A strength to this study is its originality in terms of exploring treatment-experiences from ED patients who have dropped out from a particular treatment. Adding credibility to the findings is the fact that the interviewer had no prior relation to the participants or in the developing and implementation of the PED-t. In addition, the data analysis and interpretations were conducted with a user involvement approach. A possible limitation was the sample size. The fact that only five of the 15 eligible patients were willing to participate may have restricted the variety of experiences.

### Conclusion

Our findings point to general challenges in developing procedures to explore treatment suitability, as well as capturing diversities between pre-treatment expectations and treatment content. Such procedures are relevant to promote general treatment effect factors like treatment alliance and group cohesion. To include experiences from patients who dropped out has added variety to previous studies of patient experiences with the PED-t treatment (Bakland et al., 2019; Pettersen et al., 2017). Moreover, the findings have contributed to the chain of clinical evidence of the PED-t and may lead to important treatment modifications in order to improve the treatment and thereby reduce drop out.
